# Demographic, clinical, and epidemiologic characteristics of persons under investigation for Coronavirus Disease 2019—United States, January 17–February 29, 2020

**DOI:** 10.1371/journal.pone.0249901

**Published:** 2021-04-15

**Authors:** Olivia L. McGovern, Mark Stenger, Sara E. Oliver, Tara C. Anderson, Cheryl Isenhour, Matthew R. Mauldin, Nia Williams, Eric Griggs, Tonny Bogere, Chris Edens, Aaron T. Curns, Joana Y. Lively, Yingtao Zhou, Songli Xu, Maureen H. Diaz, Jessica L. Waller, Kevin R. Clarke, Mary E. Evans, Elisabeth M. Hesse, Sapna Bamrah Morris, Robert P. McClung, Laura A. Cooley, Naeemah Logan, Andrew T. Boyd, Allan W. Taylor, Kristina L. Bajema, Stephen Lindstrom, Christopher A. Elkins, Christopher Jones, Aron J. Hall, Samuel Graitcer, Alexandra M. Oster, Alicia M. Fry, Marc Fischer, Laura Conklin, Runa H. Gokhale

**Affiliations:** 1 CDC COVID-19 Response Team, Centers for Disease Control and Prevention, Atlanta, Georgia, United States of America; 2 Epidemic Intelligence Service, Centers for Disease Control and Prevention, Atlanta, Georgia, United States of America; 3 IHRC Inc., Contracting Agency to the Centers for Disease Control and Prevention, Atlanta, Georgia, United States of America; 4 Maximus Federal, Contracting Agency to the Centers for Disease Control and Prevention, Atlanta, Georgia, United States of America; Humanitas University, ITALY

## Abstract

**Background:**

The Coronavirus Disease 2019 (COVID-19) pandemic, caused by Severe Acute Respiratory Syndrome Coronavirus 2 (SARS-CoV-2), evolved rapidly in the United States. This report describes the demographic, clinical, and epidemiologic characteristics of 544 U.S. persons under investigation (PUI) for COVID-19 with complete SARS-CoV-2 testing in the beginning stages of the pandemic from January 17 through February 29, 2020.

**Methods:**

In this surveillance cohort, the U.S. Centers for Disease Control and Prevention (CDC) provided consultation to public health and healthcare professionals to identify PUI for SARS-CoV-2 testing by quantitative real-time reverse-transcription PCR. Demographic, clinical, and epidemiologic characteristics of PUI were reported by public health and healthcare professionals during consultation with on-call CDC clinicians and subsequent submission of a CDC PUI Report Form. Characteristics of laboratory-negative and laboratory-positive persons were summarized as proportions for the period of January 17−February 29, and characteristics of all PUI were compared before and after February 12 using prevalence ratios.

**Results:**

A total of 36 PUI tested positive for SARS-CoV-2 and were classified as confirmed cases. Confirmed cases and PUI testing negative for SARS-CoV-2 had similar demographic, clinical, and epidemiologic characteristics. Consistent with changes in PUI evaluation criteria, 88% (13/15) of confirmed cases detected before February 12, 2020, reported travel from China. After February 12, 57% (12/21) of confirmed cases reported no known travel- or contact-related exposures.

**Conclusions:**

These findings can inform preparedness for future pandemics, including capacity for rapid expansion of novel diagnostic tests to accommodate broad surveillance strategies to assess community transmission, including potential contributions from asymptomatic and presymptomatic infections.

## Introduction

The pandemic of Coronavirus Disease 2019 (COVID-19), caused by Severe Acute Respiratory Syndrome Coronavirus 2 (SARS-CoV-2), evolved rapidly after the first cases were reported in Wuhan, Hubei Province, China, in December 2019. By February 29, 2020, 85,403 confirmed COVID-19 cases were reported globally: 66,337 from Hubei Province, China, 13,057 from other provinces in China, and 6,009 from 53 other countries [1]. During February, COVID-19 transmission globally was shifting from importation by travelers from China to sustained within-country community transmission and importation from countries other than China [2]. By March 11, 2020, COVID-19 had been detected in 113 countries and the World Health Organization (WHO) declared the COVID-19 outbreak a global pandemic [3]. Aggressive containment measures were implemented in the United States to slow COVID-19 introduction, including screening travelers entering the United States, restrictions on international travel outbound from the United States, quarantine of persons thought to be exposed to COVID-19, isolation of laboratory-confirmed COVID-19 cases, and contact tracing [4, 5].

In the early stages of the pandemic, the U.S. Centers for Disease Control and Prevention (CDC) worked with state, local, and territorial partners to identify and test persons thought to be at risk for COVID-19 using the CDC COVID-19 persons under investigation (PUI) evaluation criteria (Table 1) [6]. This report describes demographic, clinical, and epidemiologic characteristics among 544 PUI reported to the CDC during January 17–February 29, 2020, including 36 confirmed COVID-19 cases.

**Table 1:** CDC COVID-19 persons under investigation evaluation criteria, United States, January 17–February 29, 2020^a^.

**Date**	**Signs and symptoms**^b^	**AND**	**Epidemiologic exposure**^**c**^
**January 17–31**	Fever ***AND*** Symptoms of lower respiratory tract illness	AND	History of travel from Wuhan ***OR*** Close contact with a U.S. laboratory-confirmed COVID-19 case or ill PUI
**February 1–12**	Fever ***OR*** Symptoms of lower respiratory illness	AND	Close contact with a U.S. laboratory-confirmed COVID-19 case
	Fever ***AND*** Symptoms of lower respiratory illness	AND	Travel from Hubei Province, China
	Fever ***AND*** Symptoms of lower respiratory illness requiring hospitalization	AND	Travel from mainland China
**February 13–27**^d^	Fever ***OR*** Symptoms of lower respiratory illness	AND	Close contact with a U.S. laboratory-confirmed COVID-19 case
	Fever ***AND*** Symptoms of lower respiratory illness	AND	Travel from Hubei Province, China
	Fever ***AND*** Symptoms of lower respiratory illness requiring hospitalization	AND	Travel from mainland China
**February 28–29**	Fever ***OR*** Symptoms of lower respiratory illness	AND	Close contact with a U.S. laboratory-confirmed COVID-19 case
	Fever ***AND*** Symptoms of lower respiratory illness	AND	Travel from affected geographic areas^e^
	Fever with severe acute lower respiratory illness (e.g., pneumonia, acute respiratory distress syndrome) requiring hospitalization and without alternative explanatory diagnosis (e.g., influenza)^d^	AND	No known exposure

^a^The criteria are intended to serve as guidance for evaluation. Patients were evaluated and discussed with public health departments on a case-by-case basis.

^b^Signs and symptoms of lower respiratory illness included cough, shortness of breath, tachypnea, hypoxemia, or infiltrate observed on chest x-ray.

^c^Epidemiologic exposure must have occurred within the 14 days preceding illness onset.

^d^On February 12, a modification was added in the form of a footnote; this change was for addition of a criterion for patients with severe unexplained respiratory illness, regardless of epidemiologic exposures. This modification was implemented on February 13 and added to the main body of the PUI evaluation criteria on February 28.

^e^As of February 29, affected geographic areas were China, Iran, Italy, Japan, and South Korea. Affected geographic areas expanded as the outbreak evolved.

## Materials and methods

### Study design and participants

The surveillance cohort consisted of PUI reported to CDC by public health officials and healthcare personnel during January 17–February 29, 2020. CDC on-call clinicians provided consultation to state, local, and territorial health department officials and healthcare personnel to make case-by-case determinations of COVID-19 risk and facilitated subsequent testing for SARS-CoV-2.

PUI determination and SARS-CoV-2 testing recommendation was guided by the CDC COVID-19 PUI evaluation criteria (Table 1), which were developed by CDC and subject to clinical judgement. PUI evaluation criteria changed according to the evolving understanding of signs, symptoms, and epidemiologic exposures thought to be associated with COVID-19. On January 31, the travel-related exposure criterion expanded from Wuhan, Hubei Province, China, to all regions of China [6]. On February 12, a criterion was added as a footnote for patients with severe unexplained respiratory illness, regardless of epidemiologic exposures. On February 28, the travel-related exposure criterion was further expanded to include any geographic area affected by COVID-19, and the criterion for patients with severe unexplained respiratory illness, regardless of epidemiologic exposures, was added to the main body of the listed criteria [7]. After further local investigation, some persons initially recommended for SARS-CoV-2 testing were ultimately determined to not be PUI by state, local, or territorial public health officials for various reasons, including alternative explanation for symptoms or lack of known exposures. No SARS-CoV-2 testing was performed for these individuals.

Respiratory specimens were collected from all PUI for SARS-CoV-2 testing (i.e., nasopharyngeal and oropharyngeal swabs for all, sputum for PUI with productive cough, and bronchoalveolar lavage if clinically indicated). PUI identified in association with the SARS-CoV-2 outbreak on the Diamond Princess cruise ship were expected to have unique epidemiologic characteristics in comparison to other PUI and therefore were excluded from this analysis [8].

### Data collection

State, local, and territorial health department officials and healthcare personnel from local hospitals reported demographic, clinical, and epidemiologic data on PUI during consultation with on-call CDC clinicians and by subsequent submission of a CDC PUI Report Form [9]. Data elements included sex, age, healthcare personnel status, travel history, close contact with U.S. laboratory-confirmed COVID-19 cases, hospitalization, presenting signs and symptoms, and diagnoses for pneumonia or acute respiratory distress syndrome.

When possible, CDC data managers contacted public health professionals or healthcare personnel initially reporting PUI to complete missing data elements. Discrepancies between consultation call data and CDC PUI Report Form data were addressed through consultation with submitting health departments; when this was not possible, the source most directly connected to the PUI was used.

### Quantitative real-time reverse transcription PCR for SARS-CoV-2 detection and reporting

All SARS-CoV-2 diagnostic testing was performed using quantitative real-time reverse-transcription PCR (RT-qPCR) as described previously [10]. Data for laboratory testing results reported to CDC as of March 9 were included in this analysis.

CDC laboratories performed nearly all SARS-CoV-2 testing during the surveillance period and reported results to the CDC PUI Team. Between February 6, 2020 and February 28, 2020, two states were performing SARS-CoV-2 testing independent of CDC. From February 28, 2020 to March 2, 2020, 35 laboratories in 26 U.S. states began performing SARS-CoV-2 testing independently; for these samples, only positive results were reported to CDC. Laboratory results were considered missing if testing was performed locally and a result was not communicated to CDC or if inconsistent or missing information in linking variables did not allow for matching between CDC laboratory and epidemiologic databases.

### Data analysis

Individuals determined to be a PUI from January 17–February 29, 2020 were included in this analysis. The presence of symptoms was indicated by a check box on data collection instruments; a checked box indicated the symptom was reported whereas an unchecked box indicated that the symptom was either not reported or missing. All analyses were conducted as “present” or “no/missing”. All PUI were classified by case type and test status. Community spread was defined as PUI with neither travel- nor contact-related exposures reported. Characteristics of laboratory-negative PUI and confirmed COVID-19 cases were summarized as percentage or means and ranges for the period of January 17−February 29. All PUI with laboratory results available were compared before and after February 12, which corresponded to a PUI evaluation criteria change and approximated the shift in detected epidemiologic exposures from primarily travel-related exposure with travel from China to a greater degree of community spread within the United States and travel-related exposures from countries other than China. Prevalence ratios (PR) were calculated for comparison of categorical variables between these two time periods, and pooled t-tests were used to assess difference in means for continuous data. All data analyses were performed in SAS 9.4 (SAS Institute, Cary, NC). The CDC Human Research Protection Office determined that this work was exempt from human subjects research regulations since all information was collected in response to a public health emergency. Data were collected in compliance with applicable Office of Management and Budget approvals (OMB Control # 0920–1011).

## Results

**Fig 1:**
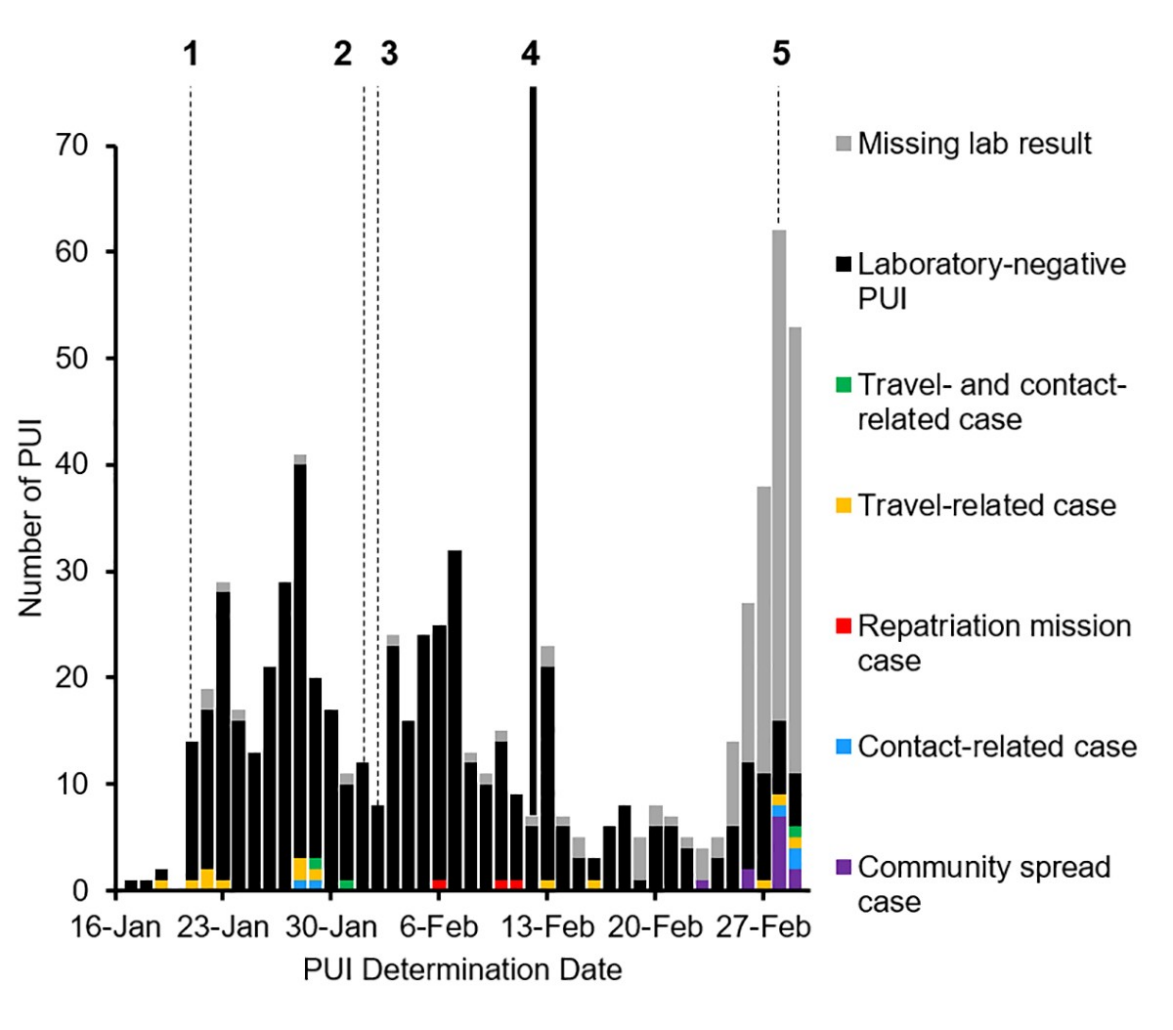
Epidemiologic curve of persons under investigation for COVID-19: Frequency by determination date, SARS-CoV-2 test status, and COVID-19 case type, United States, January 17–February 29, 2020 (*N* = 711). 1: CDC activates the Emergency Operations Center. 2: WHO declares COVID-19 outbreak a public health emergency of international concern, and persons under investigation (PUI) evaluation criteria change to expand the travel-related epidemiologic exposure criterion (Table 1). 3: The United States introduces travel restrictions for foreign nationals with travel from China in the last 14 days. 4: PUI evaluation criteria changed to include a criterion for testing of persons with severe unexplained respiratory illness by addition of a footnote. 5: PUI evaluation criteria changed to expand the travel-related exposure criterion to include any geographic area affected by COVID-19, and the criterion for patients with severe unexplained respiratory illness, regardless of epidemiologic exposures, was moved into the main body of the PUI evaluation criteria. “Missing” = testing was performed locally and a result was not communicated to CDC, or inconsistent or missing information in linking variables did not allow for matching between CDC laboratory and epidemiologic databases. Community spread cases had no known travel- or contact-related exposures.

From January 17−February 29, 2020, CDC received 2,192 consultation calls, from which 765 potential PUI were identified. Among these, 54 (7%) were determined not to be PUI after further local investigation, leaving 711 PUI with submitted specimens for testing (Fig 1). An additional 167 (22%) had missing SARS-CoV-2 RT-qPCR test results as of March 9. The 544 PUI with test results reported to CDC that could be matched to PUI epidemiologic data represent patients from 42 U.S. states and the U.S. Virgin Islands; among these, 36 (7%) were confirmed COVID-19 cases.

Overall, laboratory-negative PUI and confirmed COVID-19 cases presented with similar demographic characteristics (Table 2). Proportions of males and females were similar for both confirmed COVID-19 cases and laboratory-negative PUI. Confirmed COVID-19 cases were slightly older compared to laboratory-negative PUI during this time period (mean in years [range]: 46 [15–77] versus 37 [<1–92]). Among 62 PUI under age 18 years tested for SARS-CoV2; 6 (10%) were positive. Among 67 healthcare personnel tested for SARS-CoV-2, 2 (3%) were positive, and neither had known exposure in a U.S. healthcare setting.

**Table 2:** Characteristics of U.S. laboratory-negative persons under investigation and confirmed COVID-19 cases, January 17–February 29, 2020^a^.

Characteristic	Laboratory-negative PUI, n = 508	Confirmed COVID-19 cases, n = 36
Sex, no. (%)		
M	271 (53.4)	17 (47.2)
F	237 (46.7)	17 (47.2)
Unknown	0 (0.0)	2 (5.9)
Age		
Age <18 years, no. (%)	56 (11)	6 (16.7)
Age in years, mean (range)	37.3 (1–92)	46.1 (15–77)
Healthcare personnel, no. (%)	65 (12.8)	2 (5.6)
Hospitalized at PUI report, no. (%)^b^	144 (28.4)	16 (44.4)
Presenting symptoms, no. (%)		
Any	501 (98.6)	30 (83.3)
Shortness of breath	140 (27.6)	10 (27.8)
Objective or subjective fever	344 (67.7)	21 (58.3)
Mean temperature for objective fever	101.2	101.6
Sore throat	207 (40.7)	11 (30.6)
Gastrointestinal symptoms^c^	102 (20.1)	3 (8.3)
Mean temperature for objective fever, °F	101.2	101.6
Signs and clinical diagnoses, no. (%)		
Any signs	354 (69.9)	24 (66.7)
Abnormal chest x-ray	38 (7.5)	5 (13.9)
Pneumonia diagnosed (clinical or radiologic)	51 (10.0)	5 (13.9)
Acute respiratory distress diagnosed	11 (2.2)	2 (5.6)
Epidemiologic exposures, no. (%)		
Any travel-related exposure^d^	392 (77.2)	19 (52.8)
Travel from Hubei Province, China^e^	186 (36.6)	12 (33.3)
Other travel from China (not Hubei)	170 (33.5)	3 (8.3)
Travel from countries other than China	36 (7.1)	4 (11.1)
Contact with a U.S. confirmed COVID-19 case^d^^,^^f^	109 (21.4)	8 (22.2)
Both travel- and contact-related exposures	30 (5.9)	3 (8.3)
No known travel- or contact-related exposure	36 (7.1)	12 (33.3)

^a^ Table 2 includes only persons under investigation (PUI) who had complete COVID-19 test results reported to the CDC. This included repatriation missions from Hubei Province, China but not the Diamond Princess cruise ship.

^b^ Most patients were hospitalized for severity of illness, but some were hospitalized for isolation purposes.

^c^ Gastrointestinal symptoms included nausea, vomiting, diarrhea, and abdominal pain. The confirmed COVID-19 case reporting gastrointestinal symptoms reported abdominal pain.

^d^ Includes the 33 persons with both travel- and contact-related exposures.

^e^ Includes persons on Wuhan repatriation flights. There were 25 laboratory-negative PUI and 3 confirmed COVID-19 cases with travel from Hubei Province on Wuhan repatriation flights.

^f^ Asymptomatic contacts under investigation were not included in this analysis.

Laboratory-negative PUI and confirmed COVID-19 cases also showed similar clinical presentation at the time of PUI report; however, a higher proportion of confirmed COVID-19 cases were hospitalized compared to laboratory-negative PUI (44% [16/36] versus 28% [144/508]). Consistent with the PUI evaluation criteria, confirmed COVID-19 cases most commonly presented with cough (75% [27/36]) or fever (58% [21/36]), and less commonly with shortness of breath (28% [10/36]), sore throat (31% [11/36]), or gastrointestinal symptoms (8% [3/36]).

Overall, a similar proportion of laboratory-negative PUI and confirmed COVID-19 cases reported travel from Hubei Province, China; travel from countries other than China; and contact with a U.S. confirmed COVID-19 case (Table 2). However, compared to laboratory-negative PUI, a higher proportion of confirmed COVID-19 cases traveled from Hubei Province, China, than from other provinces of China (33% [12/36] versus 8% [3/36]). Among the 36 confirmed COVID-19 cases, 16 (44%) reported only travel, 5 (14%) reported only being contacts of a confirmed case, 3 (8%) reported both travel and being a contact, and 12 (33%) reported no known exposures, indicating possible community spread.

A comparison of the demographic, clinical, and epidemiologic characteristics of all PUI before and after the February 12 PUI evaluation criteria change is presented in Table 3. Consistent with changes in PUI evaluation criteria, PUI reported after February 12 were less likely to report travel from Hubei Province, China (PR: 0.2, p<0.001) and more likely to report either travel from a country other than China (PR: 4.6, p<0.001) or having no known travel- or contact-related exposures (PR: 3.7, p<0.001). PUI reported after February 12 also tended to be older (mean in years [range]: 43 [2–87] versus 36 [<1–92]) and more likely to report shortness of breath (PR: 1.7, p<0.001), but they were less likely to report cough (PR: 0.6, p<0.05) or fever (PR: 0.7, p<0.05). A higher proportion of PUI were confirmed COVID-19 cases after February 12 (PR: 2.8, p<0.001).

**Table 3:** Characteristics of persons under investigation for COVID-19, United States, January 17–February 12 and February 13–29, 2020.

Characteristic	January 17–February 12, n = 420	February 13–February 29, n = 124	Prevalence ratio (PR) (95% CI) or t-test *P*-value
Sex, no. (%)			
Male	229 (54.5)	59 (47.6)	0.80 (0.6–1.1)
Female	191 (45.5)	63 (50.8)	
Unknown Sex	0 (0.0)	2 (1.6)	
Age in years, mean (range)	36.4 (1–92)	43 (2–87)	0.0005
Healthcare personnel, no. (%)	49 (11.7)	18 (14.5)	1.2 (0.8–1.9)
Epidemiologic exposures, no. (%)			
Any travel-related exposure^c^	329 (78.3)	83 (66.9)	0.8 (0.6–1.2)
Travel from Hubei Province	182 (43.3)	16 (12.9)	0.25 (0.16–0.43)^a^
Other travel from China (not Hubei)	140 (33.3)	33 (26.6)	0.78 (0.55–1.11)
Travel from countries other than China	7 (1.6)	34 (27.4)	4.6 (3.7–5.9)^a^
Contact with a U.S. confirmed COVID-19 case^c^	105 (25.0)	12 (9.6)	0.5 (0.3–1.0)
Both travel- and contact-related exposure	29 (6.9)	4 (3.2)	0.5 (0.2–1.3)
No known travel- or contact-related exposure	15 (3.6)	33 (26.6)	3.7 (2.9–4.9)^a^
Presenting symptoms, no. (%)			
Cough	375 (89.3)	102 (82.3)	0.65 (0.44–0.95)^b^
Objective or subjective fever	291 (69.3)	74 (59.7)	0.73 (0.53–0.99)^b^
Sore throat	173 (42.3)	45 (36.3)	0.9 (0.62–1.17)
Shortness of breath	101 (24.1)	49 (39.5)	1.71 (1.26–2.33)^a^
Confirmed COVID-19 cases, no. (%)	15 (3.6)	21 (16.9)	2.8 (2.1–4.0)^a^

^a^p<0.001

^b^p<0.05; all others had p≥0.05.

^c^ Includes the 33 persons with both travel- and contact-related exposures.

As depicted in Fig 1, 15 confirmed COVID-19 cases were reported during January 17 to February 12: 11 (73%) reported travel-related exposure only, 2 (13%) reported contact with a U.S. confirmed COVID-19 case only, and 2 (13%) reported both travel- and contact-related exposure. All travel-related exposures during this time were travel from China including travel from Wuhan, Hubei Province, on repatriation mission flights, and 1 travel-related case was among a repatriated person who worked as a healthcare personnel in Hubei Province, China.

During February 13 to February 29, more than half of confirmed COVID-19 cases reported no known travel- or contact-related exposure and were therefore likely associated with community spread (Fig 1). A total of 21 confirmed COVID-19 cases were reported during this time: 12 (57%) were associated with community spread and identified on or after February 23; 3 (14%) reported contact with a U.S. confirmed COVID-19 case only; 5 (24%) reported travel-related exposure only; and 1 (5%) reported both travel and contact-related exposure. Travel-related cases reported travel from China, South Korea, and Egypt. One case of community spread occurred in someone identified as a U.S. healthcare personnel.

## Discussion

This report provides a description of PUI during the early stages of the COVID-19 pandemic in the United States. Overall, the demographic, clinical, and epidemiologic characteristics of laboratory-negative PUI and confirmed COVID-19 cases were similar and generally align with other reports from similar time periods. Confirmed COVID-19 cases presented commonly with cough and/or fever [11–13] and less often with shortness of breath, sore throat or gastrointestinal symptoms [11, 13]. Further, most cases occurred in middle- to older-aged adults [14]. However, 16.7% of U.S. confirmed COVID-19 cases, identified by February 29, 2020, were children under 18 years of age, which is higher than reported in China [14]. During the first few months of the U.S. pandemic, pediatric COVID-19 cases presented with less severe illness and fewer symptoms than adults [15]. Further investigation will help to understand the true range of clinical presentations and distribution of SARS-CoV-2 infection among all age groups including those with symptomatic and asymptomatic infection.

While healthcare personnel are at higher risk of exposure to COVID-19 than the general public [16], infection among healthcare personnel was relatively rare in the United States during the early stage of the pandemic. Similarly, Ghinai et al. and Heinzerling et al. found that more than half of the 331 healthcare personnel who provided care to two COVID-19 patients reported medium- or high-risk exposures to the COVID-19 patients, yet only 3 tested positive [17, 18]. As the number of cases in the United States has risen, so too has the number of healthcare personnel affected [19]. Additional studies of healthcare personnel will elucidate the circumstances and settings that facilitate or protect against SARS-CoV-2 transmission.

Finally, this report describes a shift in detected epidemiologic exposures before and after February 12, 2020, corresponding to a change in the PUI evaluation criteria in the United States. Before February 12, epidemiologic exposures among PUI were primarily travel-related with travel from China. After February 12, a greater proportion of PUI had either travel-related exposures with travel from countries other than China or no known travel- or contact-related exposures. While these shifts in detected epidemiologic exposures likely reflected in part the global spread of COVID-19, including more community transmission within the United States, these results were also likely driven by travel restrictions and changes in PUI evaluation criteria made during this period. Beginning February 2, travel restrictions were put into place that denied entry into the U.S. for foreign nationals with travel from China in the 14 days before their arrival, and expansion of the travel-related exposure criterion to any geographic area affected by COVID-19 was added to the PUI evaluation criteria on February 28. In addition, an official criterion for patients with severe unexplained respiratory illness, regardless of epidemiologic exposure, was added to the PUI evaluation criteria as a footnote on February 12 and into the main body of the PUI evaluation criteria on February 28. While the criterion may not have been widely recognized prior to its addition into the main body of the PUI criteria, deferral to clinical judgment allowed for testing of persons not fully meeting the PUI evaluation criteria throughout this time period.

A better understanding of when and how the pandemic shifted from primarily travel-related cases to community transmission in the United States, including work such as that undertaken by Jorden and colleagues demonstrating limited early spread [20], will benefit future planning for other novel pathogens with rapidly changing epidemiology. Several factors likely contributed to the rapid acceleration of the COVID-19 pandemic in the United States, including cryptic transmission within the community due to limited laboratory testing and spread by persons with asymptomatic or presymptomatic infection [21]. While PUI evaluation criteria changed as our understanding of COVID-19 epidemiology evolved, the criteria guided detection of persons with known epidemiologic exposures and signs and symptoms of lower respiratory illness. Knowledge of transmission by asymptomatic and presymptomatic persons was limited early in the pandemic; therefore, they were not targeted for testing during this time. In addition, there was limited testing in the early phases of the outbreak, focused primarily on those with severe illness or PUI thought to have known high-risk epidemiologic exposures. Taken together, these factors likely contributed to underdetection of COVID-19 early in the pandemic.

This analysis is subject to several limitations. Evaluation of PUI was based on patient presentation at the time of consultation with CDC’s PUI clinical call team; no follow-up data were received. Further, patients with atypical presentations not included in the PUI evaluation criteria such as GI illness without fever and cough may not be represented in this analysis. Therefore, the full spectrum of COVID-19 clinical illness may not be represented in these data. PUI who tested negative for SARS-CoV-2 might have been underreported during the last week of the surveillance period, since 26 jurisdictions began testing independently but were submitting reports only for PUI who tested positive for SARS-CoV-2. All missing data for variables recorded using check boxes were treated as a “no” response, which might lead to underestimation of prevalence if some were unknown or truly missing responses. This report includes a relatively small number of confirmed COVID-19 cases, which may not be generalizable to later cases, especially given the ongoing evolution of COVID-19 epidemiology in the United States and globally.

## Conclusions

Infectious disease outbreaks with a central geographic locus allow for targeted intervention such as travel restrictions and testing of persons with defined epidemiologic links. However, given the nature of global travel, widespread dissemination to a global pandemic can occur rapidly. An understanding of the beginning stages of the pandemic within the United States, can provide insight into its ultimate evolution toward widespread community transmission. The description of this early response to COVID-19 within the United States can inform future pandemic preparedness planning, including preparation of healthcare systems and public health laboratories for early and rapid expansion of testing for novel pathogens, implementation of broader testing criteria, and early testing of asymptomatic contacts.

## References

[1] World Health Organization. Coronavirus disease 2019 (COVID-19) situation report—40. 2020 3 4 [cited 2020 Mar 30]. https://www.who.int/docs/default-source/coronaviruse/situation-reports/20200304-sitrep-44-covid-19.pdf?sfvrsn=93937f92_6

[2] World Health Organization. Coronavirus disease 2019 (COVID-19) situation report—38. 2020 2 27 [cited 2020 Mar 30]. https://www.who.int/docs/default-source/coronaviruse/situation-reports/20200227-sitrep-38-covid-19.pdf?sfvrsn=2db7a09b_4

[3] World Health Organization. Coronavirus disease 2019 (COVID-19) situation report—51. 2020 3 11 [cited 2020 Mar 30]. https://www.who.int/docs/default-source/coronaviruse/situation-reports/20200311-sitrep-51-covid-19.pdf?sfvrsn=1ba62e57_10

[4] Burke RM, Midgley CM, Dratch A, Fenstersheib M, Haupt T, Holshue M, et al. Active monitoring of persons exposed to patients with confirmed COVID-19—United States, January–February 2020. MMWR Morb Mortal Wkly Rep. 2020;69:245–6. 10.15585/mmwr.mm6909e1 32134909 PMC7367094

[5] Jernigan DB; CDC COVID-19 Response Team. Update: public health response to the coronavirus disease 2019 outbreak—United States, February 24, 2020. MMWR Morb Mortal Wkly Rep. 2020;69:216–9. 10.15585/mmwr.mm6908e1 32106216 PMC7367075

[6] Bajema KL, Oster AM, McGovern OL, Lindstrom S, Stenger MR, Anderson TC, et al. Persons evaluated for 2019 novel coronavirus—United States, January 2020. MMWR Morb Mortal Wkly Rep. 2020;69:166–70. 10.15585/mmwr.mm6906e1 32053579 PMC7017962

[7] Centers for Disease Control and Prevention. Update and Interim Guidance on Outbreak of Coronavirus Disease 2019 (COVID-19). 2020;CDCHAN-00428. 2020 2 28 [cited 2020 May 22]. https://emergency.cdc.gov/han/2020/han00428.asp

[8] Moriarty LF, Plucinski M, Marston BJ, Kurbatova EV, Knust B, Murray EL, et al. Public health responses to COVID-19 outbreaks on cruise ships—Worldwide, February–March 2020. MMWR Morb Mortal Wkly Rep. 2020;69:347–52. 10.15585/mmwr.mm6912e3 32214086 PMC7725517

[9] Centers for Disease Control and Prevention. Information for health departments on reporting a person under investigation (PUI), or presumptive positive and laboratory-confirmed cases of COVID-19. 2020 3 29 [cited 2020 Mar 30]. https://www.cdc.gov/coronavirus/2019-ncov/php/index.html

[10] Lu XW L.; Sakthivel S.K.; Whitaker B.; Murray J.; Kamili S.; et al. US CDC real-time reverse transcription PCR panel for detection of severe acute respiratory syndrome coronavirus 2. Emerg Infect Dis. 2020;2020 8 [cited 2020 June 11]. 10.3201/eid2608.201246 PMC7392423 32396505

[11] Wang D, Hu B, Hu C, Zhu F, Liu X, Zhang J, et al. Clinical Characteristics of 138 Hospitalized Patients With 2019 Novel Coronavirus-Infected Pneumonia in Wuhan, China. JAMA. 2020;323:1061–1069. 10.1001/jama.2020.1585 32031570 PMC7042881

[12] Wei M, Yuan J, Liu Y, Fu T, Yu X, Zhang ZJ. Novel Coronavirus Infection in Hospitalized Infants Under 1 Year of Age in China. JAMA. 2020;323:1313–1314. 10.1001/jama.2020.2131 32058570 PMC7042807

[13] Zhang JJ, Dong X, Cao YY, Yuan YD, Yang YB, Yan YQ, et al. Clinical characteristics of 140 patients infected with SARS-CoV-2 in Wuhan, China. Allergy. 2020 2 19. 10.1111/all.14238 32077115

[14] Liu Z, Bing, Zhi XZ, Epidemiology Working Group for NCIP Epidemic Response, Chinese Center for Disease Control and Prevention. The epidemiological characteristics of an outbreak of 2019 novel coronavirus diseases (COVID-19) in China [in Mandarin]. 2020;41:145–51.

[15] Team CC-R. Coronavirus Disease 2019 in Children—United States, February 12-April 2, 2020. MMWR Morb Mortal Wkly Rep. 2020;69:422–426. 10.15585/mmwr.mm6914e4 32271728 PMC7147903

[16] Nguyen LH, Drew DA, Graham MS, Joshi AD, Guo C, Ma W, et al. Risk of COVID-19 among front-line health-care workers and the general community: a prospective cohort study. Lancet Public Health. 2020;5;e475–e483. 10.1016/S2468-2667(20)30164-X 32745512 PMC7491202

[17] Ghinai I, McPherson TD, Hunter JC, Kirking HL, Christiansen D, Joshi K, et al. First known person-to-person transmission of severe acute respiratory syndrome coronavirus 2 (SARS-CoV-2) in the USA. Lancet. 2020;395:1137–1144. 10.1016/S0140-6736(20)30607-3 32178768 PMC7158585

[18] Heinzerling A, Stuckey MJ, Scheuer T, Xu k, Perkins KM, Resseger H, et al. Transmission of COVID-19 to Health Care Personnel During Exposures to a Hospitalized Patient—Solano County, California, February 2020. MMWR Morb Mortal Wkly Rep. 2020;69:472–476. 10.15585/mmwr.mm6915e5 32298249 PMC7755059

[19] CDC COVID-19 Response Team. Characteristics of Health Care Personnel with COVID-19—United States, February 12-April 9, 2020. MMWR Morb Mortal Wkly Rep. 2020;69:477–481. 10.15585/mmwr.mm6915e6 32298247 PMC7755055

[20] Team CC-R, Jorden MA, Rudman SL, et al. Evidence for Limited Early Spread of COVID-19 Within the United States, January-February 2020. MMWR Morb Mortal Wkly Rep. 2020;69:680–684. 10.15585/mmwr.mm6922e1 32497028 PMC7315848

[21] Schuchat A, Public Health Response to the Initiation and Spread of Pandemic COVID-19 in the United States, February 24-April 21, 2020. MMWR Morb Mortal Wkly Rep. 2020;69:551–556. 10.15585/mmwr.mm6918e2 32379733 PMC7737947

